# Exposure of Salmonella enterica Serovar Typhimurium to Three Humectants Used in the Food Industry Induces Different Osmoadaptation Systems

**DOI:** 10.1128/AEM.01379-15

**Published:** 2015-09-04

**Authors:** Sarah Finn, Lisa Rogers, Kristian Händler, Peter McClure, Alejandro Amézquita, Jay C. D. Hinton, Séamus Fanning

**Affiliations:** a UCD Centre for Food Safety, School of Public Health, Physiotherapy & Population Science, University College Dublin, Belfield, Dublin, Ireland; b Conway Institute, UCD School of Biomolecular & Biomedical Science, University College Dublin, Belfield, Dublin, Ireland; c Moyne Institute of Preventive Medicine, Trinity College Dublin, Dublin, Ireland; d Unilever, Safety and Environmental Assurance Centre, Sharnbrook, Bedfordshire, United Kingdom; e Institute of Integrative Biology, University of Liverpool, Liverpool, United Kingdom; f Institute for Global Food Security, School of Biological Sciences, Queen's University Belfast, Belfast, Northern Ireland

## Abstract

Common salt (NaCl) is frequently used by the food industry to add flavor and to act as a humectant in order to reduce the water content of a food product. The improved health awareness of consumers is leading to a demand for food products with reduced salt content; thus, manufacturers require alternative water activity-reducing agents which elicit the same general effects as NaCl. Two examples include KCl and glycerol. These agents lower the water activity of a food matrix and also contribute to limit the growth of the microbiota, including foodborne pathogens. Little is currently known about how foodborne pathogens respond to these water activity-lowering agents. Here we examined the response of Salmonella enterica serovar Typhimurium 4/74 to NaCl, KCl, and glycerol at three time points, using a constant water activity level, compared with the response of a control inoculum. All conditions induced the upregulation of gluconate metabolic genes after 6 h of exposure. Bacteria exposed to NaCl and KCl demonstrated the upregulation of the osmoprotective transporter mechanisms encoded by the *proP*, *proU*, and *osmU* (STM1491 to STM1494) genes. Glycerol exposure elicited the downregulation of these osmoadaptive mechanisms but stimulated an increase in lipopolysaccharide and membrane protein-associated genes after 1 h. The most extensive changes in gene expression occurred following exposure to KCl. Because many of these genes were of unknown function, further characterization may identify KCl-specific adaptive processes that are not stimulated by NaCl. This study shows that the response of *S*. Typhimurium to different humectants does not simply reflect reduced water activity and likely involves systems that are linked to specific humectants.

## INTRODUCTION

Addition to foods of compounds known as humectants that function to control water activity (*a_w_*) is an age-old process of enhancing stability, adding flavor, and limiting food spoilage. The oldest and most widely used of these humectants is common salt (NaCl) and various sugars, such as sucrose and fructose. Other humectants that can be used include glycerol, sorbitol, and KCl. Many Gram-negative bacteria, including Salmonella, require an *a_w_* of >0.93 for growth, with optimum growth occurring at *a_w_*s of 0.995 to 0.98 (1, 2). However, this pathogen has the ability to persist within food production environments, wherein the water activity levels are often carefully controlled. Further, Salmonella is the etiological agent in the majority of outbreaks linked to low-water-activity foods (3–5). Some studies have begun to shed light on the mechanisms by which Salmonella survives within a dry factory environment (6–8). Similarly, a study of the response(s) of Salmonella in a low-water-activity food product reported that the bacteria enter a dormant state during which <5% of the genome is transcribed (9).

When bacterial cells sense a decrease in the moisture availability of the external environment, they must respond rapidly to balance the internal osmotic pressure in order to maintain viability. Initially, it was believed that the type of response was not dependent on the nature of the osmolyte and that an increase in potassium influx was a common feature of adaption (10–12). However, Shabala et al. demonstrated that the osmotic response of Escherichia coli is highly dependent on the nature of the solute (13). In the latter investigation, the response of E. coli to NaCl and sucrose was examined after 10 min, with 40% of the differentially expressed genes sharing no similarity between the two solutes studied (13). These authors concluded that osmotic challenge, whether it is from ionic or nonionic humectants, can elicit rather different responses (13).

The response elicited by Gram-negative bacteria upon exposure to elevated levels of NaCl has been a topic of investigation for many years (14–18). Due to the harmful health effects (such as elevated blood pressure and cardiovascular disease) associated with high levels of NaCl consumed in the diet, improved consumer awareness of these negative health effects has resulted in the demand for products with much lower salt contents, and this consumer-driven pressure has led manufacturers to seek out alternative humectants. Two such alternative compounds are KCl and glycerol. The transcriptional mechanisms by which Salmonella responds to the stress exerted by these humectant compounds have not been thoroughly examined.

Both NaCl and KCl are classified as ionic humectant compounds; hence, it is hypothesized that the bacterial transcriptional response to these chemicals may be very similar. In contrast, glycerol is a nonionic sugar alcohol (polyol) that freely permeates the cytoplasmic membrane (19). How Salmonella responds to glycerol-induced low-water-activity conditions is unknown.

The aim of this study was to examine the transcriptional response of Salmonella enterica serovar Typhimurium 4/74 to the three humectants NaCl, KCl, and glycerol while the water activity level was maintained at a sublethal level (*a_w_* = 0.95) over a 24-h period. This particular strain was chosen for analysis as it has been widely used as a model bacterium in the context of transcriptomic analysis, allowing comparison of the data obtained by transcriptomic analysis with data obtained by other methods, such as by desiccation (6), and under other infection-relevant conditions (20). These data may help food manufacturers develop and validate improved food safety measures for low-moisture foods.

## MATERIALS AND METHODS

### Bacterial strains and inoculum preparation.

*S*. Typhimurium 4/74 was used throughout this study (21). This isolate was stored on cryobeads (Technical Service Consultants Ltd., Heywood, Lancashire, England) at −80°C, resuscitated from storage directly on Luria-Bertani (LB) agar (Difco), and incubated overnight at 37°C.

Similar to the experiments described previously by Finn et al., a setting with a static system of growth at 24°C was used throughout the study to model a storage setting that may be encountered by these bacteria within a low-moisture food and its production setting (6). A standard early-stationary-phase (ESP) inoculum was prepared in LB medium using a previously described method (6). Briefly, 10 ml of LB medium was inoculated using one colony from an LB agar plate and incubated statically at 24°C for 48 h. The culture was then diluted 1:100, and 200 μl of this cell suspension was used to inoculate 500 ml LB medium to yield approximately 4 × 10^3^ CFU/ml. The culture was incubated statically at 24°C until it reached early stationary phase (approximately 17 h) and served as the inoculum for further study.

### Exposure to humectants and RNA extraction.

The MICs of NaCl, KCl, and glycerol for isolate 4/74 were previously determined (22). On the basis of these results, sublethal concentrations of all three compounds were prepared in Luria-Bertani broth without NaCl (LO; 10 g/liter tryptone, 5 g/liter yeast extract) as follows: 6% (wt/vol) NaCl (*a_w_* = 0.951), 9.25% (wt/vol) KCl (*a_w_* = 0.953), and 10% (vol/vol) glycerol (*a_w_* = 0.951). Each of these medium preparations was measured using a LabMaster-*a_w_* meter (Novasina AG, Lanchen, Switzerland).

Samples (10 ml) of the inoculum culture were centrifuged at 3,200 × *g* for 10 min. The supernatant was discarded, and the cell pellet was washed three times with phosphate-buffered saline (PBS). Finally, the cells in the pellet were resuspended in 10 ml of NaCl, KCl, or glycerol medium and incubated statically at 24°C for 1, 6, or 24 h. These time points were chosen to examine the cellular changes that take place at a relatively early exposure (1 h) and the adaptive mechanisms that take place after a longer-term exposure (24 h).

To extract RNA from the samples indicated above, cells were harvested by centrifugation at 3,200 × *g* for 10 min; the majority of the supernatant (approximately 9 ml) was discarded and the pellet was resuspended in the remaining liquid (approximately 1 ml). The cell suspension was then transferred to a 1.5-ml microcentrifuge tube and centrifuged for 1 min at 20,800 × *g*. The supernatant was subsequently discarded, and the cell pellet was resuspended on ice in 1 ml TRIzol reagent (Invitrogen). Total RNA was extracted as described by Kröger et al. (20) and treated with DNase I as described by Finn et al. (6). RNA was adjusted to a final concentration of >1,300 ng/μl with RNase-free water, as determined using a NanoDrop ND-1000 spectrophotometer (NanoDrop, Wilmington, DE, USA).

An Agilent RNA 6000 Nano kit (catalog no. 5067-1511) was used to assess RNA quality using an Agilent 2100 bioanalyzer (Agilent, Stockport, United Kingdom) per the manufacturer's instruction.

### Microarray preparation and transcriptome analysis.

Microarray slides were prepared, scanned, and analyzed as outlined previously (6). Methods are described in brief below. A SALSIFY2 array was used in this study (Agilent microarray design identifier [AMADID] 037367; Agilent Technologies, Santa Clara, CA). A previously described common reference approach which avoids the use of dye-swap experiments and allows comparison of the data obtained under different conditions was used (23–25). In this case, *S*. Typhimurium 4/74 genomic DNA (gDNA) was used as the standard reference. RNA was reverse transcribed to cDNA and fluorescently labeled with Cy3-dCTP, while *S*. Typhimurium 4/74 gDNA was fluorescently labeled with Cy5-dCTP. The labeled gDNA and cDNA were hybridized to the array for 18 h at 65°C and washed according to the manufacturer's instructions (Agilent). Scanning was carried out using an Agilent microarray scanner (Agilent Technologies, Santa Clara, CA) at a 5-μm resolution, with the green and red photomultiplier tube (PMT) values being set to 100% and the extended-dynamic-range (XDR) value being 0.1. The data were then extracted from the resulting multi-image TIFF files using Feature Extraction software (Agilent Technologies) and analyzed using GeneSpring (version 7.3) software (Agilent Technologies, Santa Clara, CA).

The whole experiment was carried out in triplicate. Expression profiles were normalized to the profile of the inoculum ESP culture. Genes with statistically significant (*P* < 0.05) changes in expression above or below a 3-fold cutoff were identified using a *t* test. Only genes that were found by two or more probes to have changes in expression above or below the 3-fold cutoff were included in the analysis.

### qRT-PCR.

Quantitative reverse transcriptase (RT) PCR (qRT-PCR) was used for validation of the microarray results. The oligonucleotide primers used are listed in Table S1 in the supplemental material. On the basis of the results obtained from the analysis of the microarray data, two upregulated genes and two downregulated genes from each sample were selected, and the corresponding expression, normalized to the expression of the nondifferentially expressed 16S rRNA gene, was determined by qRT-PCR. Due to the degree of differential expression observed, it was not possible to use the same genes for validation across all time points. A Qiagen QuantiTect SYBR green RT-PCR kit (Qiagen, Hilden, Germany) was used for the preparation of samples, and reactions were carried out in a Mastercycler ep realplex real-time PCR system (Eppendorf, Hamburg, Germany). In brief, the amplification reaction mixtures were as follows: 12.5 μl 2× QuantiTect SYBR green RT-PCR master mix, 10 pmol of each forward and reverse primer (see Table S1 in the supplemental material), 50 ng of each RNA sample taken at a designated time, and 0.25 μl of QuantiTect reverse transcription mix, along with RNase-free water in a final volume of 25 μl. Cycling conditions were as follows: 30 min at 50°C (to allow the synthesis of cDNA) and 95°C for 15 min, followed by 40 cycles of 15 s at 94°C, 30 s at 60°C, and 30 s at 72°C. Three biological replicates were carried out, and relative gene expression was calculated using the 2^−ΔΔ*CT*^ threshold cycle (*C_T_*) method (26).

### Microarray data accession number.

Data from this study have been deposited in the NCBI Gene Expression Omnibus (GEO) database and assigned GEO accession number GSE69857.

## RESULTS

**FIG 1:**
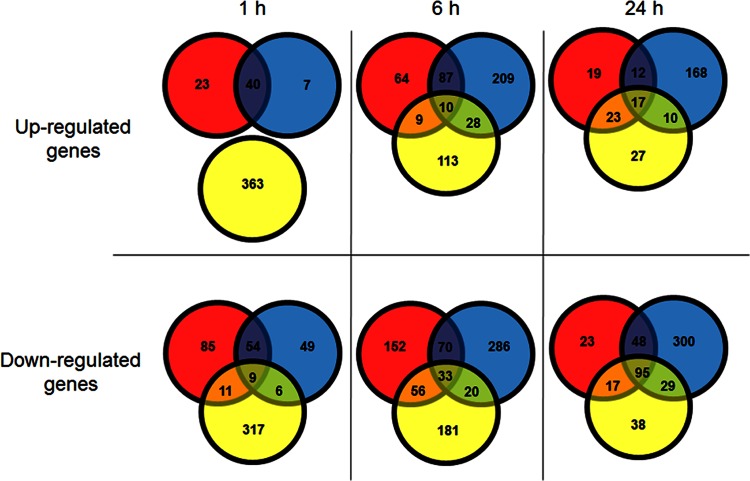
Comparison of genes differentially expressed over the course of the experiment. The numbers indicate the number of up- or downregulated genes. Red circles, NaCl-exposed samples; blue circles, KCl-exposed samples; yellow circles, glycerol-exposed samples.

A comparison of the overlap between the humectant-induced gene expression profiles is outlined in Fig. 1. Full lists of the gene expression changes detected after exposure to NaCl, KCl, and glycerol are included in Data Sets S1, S2, and S3 in the supplemental material, respectively. Table 1 outlines the total number of differentially expressed genes across all conditions. The transcriptomic data were confirmed by qRT-PCR (see Fig. S1 in the supplemental material) and showed statistically significant (*P* < 0.05) changes in the levels of expression of the genes tested compared to those for the control. The same expression patterns of the selected genes were observed both in the transcriptomic arrays and by qRT-PCR, thereby validating these data.

**TABLE 1:** Genes differentially expressed in comparison to expression of an ESP control

Time (h)	No. of genes differentially expressed in response to^*a*^:								
	NaCl			KCl			Glycerol		
	Upregulated	Downregulated	Total	Upregulated	Downregulated	Total	Upregulated	Downregulated	Total
1	63	159	222	47	118	165	363	343	706
6	170	311	481	334	409	953	160	290	450
24	71	183	254	207	472	679	77	179	256

a Differential expression was a >3-fold change with a *P* value of <0.05.

### Identification of differentially expressed genes after 1 h of exposure to the three humectants.

**FIG 2:**
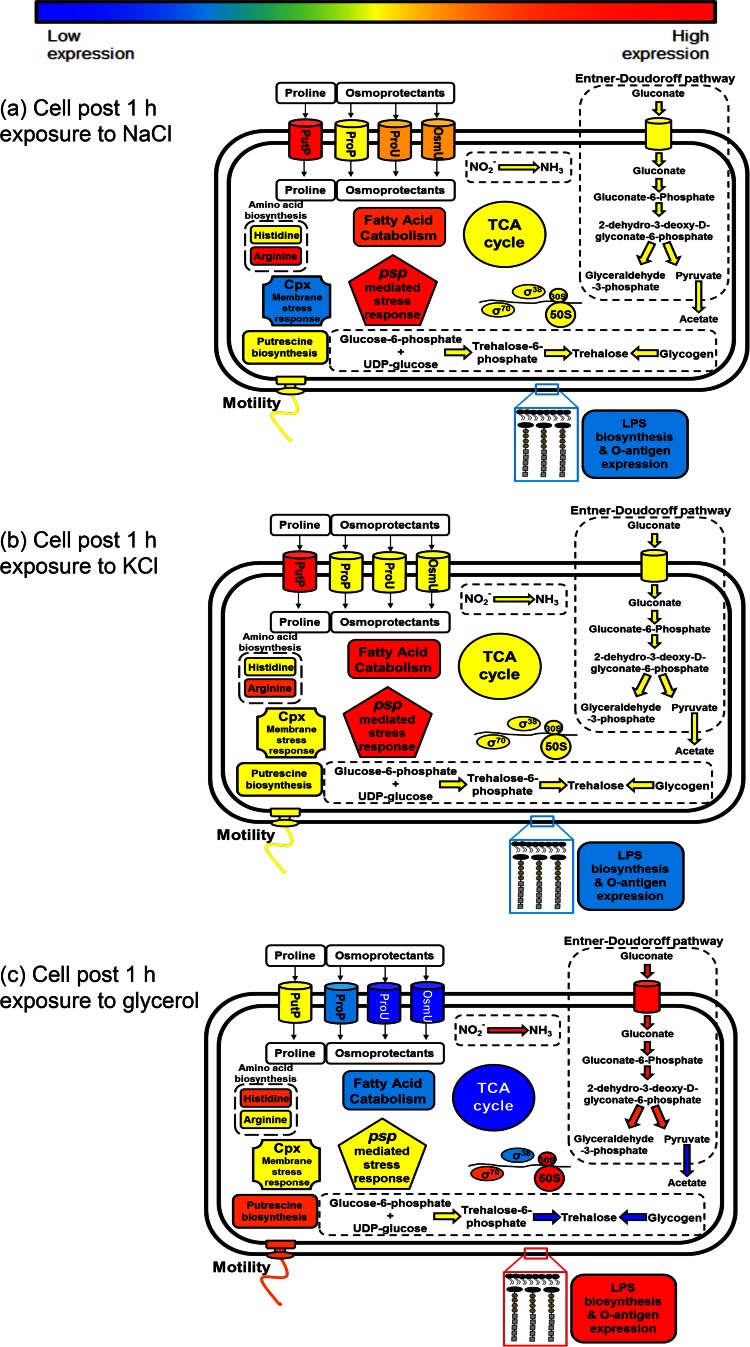
Summary of proposed changes occurring after 1 h of exposure to NaCl (a), KCl (b), or glycerol (c).

To determine the changes in gene expression relative to that of the inoculum, transcriptomic data were compared to those from an early-stationary-phase (ESP) culture. After 1 h, cells exposed to glycerol showed the most extensive changes in gene expression, with 363 genes being upregulated and 343 genes being downregulated in comparison to the gene expression profile in the control inoculum. This was followed by NaCl (with which 222 genes were differentially expressed) and, finally, KCl (with which 165 genes were differentially expressed). Interestingly, although the *a_w_* measurements of all three medium preparations were nearly identical, no upregulated genes were shared between all three conditions after 1 h of exposure. In contrast, a total of 40 of the same genes were upregulated following a 1-h exposure to NaCl and KCl. A summary of the transcriptional changes occurring in the bacterial cell at 1 h after exposure is outlined in Fig. 2.

### (i) Central metabolism and energy derivation after 1 h of exposure.

In the presence of glycerol, several changes in the expression of genes that control central metabolism occurred. Genes involved in all reactions within the tricarboxylic acid (TCA) cycle were significantly downregulated. The upregulation of both gluconate transporters (*gntT* and *gntU*) suggests that gluconate uptake is increased in response to glycerol. This is supported by the increased expression of the *gntK*, *edd*, and *eda* genes, which encode enzymes of the Entner-Doudoroff pathway, which subsequently converts gluconate to pyruvate and glyceraldehye-3-phosphate. Upregulation of the *purAB* genes could indicate an alternative method of fumarate formation mediated via the conversion of l-aspartate. Genes involved with a nitrite reductase complex (encoded by *nirB* and *nirD*) also showed increased expression, as did the nitrite transporter *nirC*, suggesting that nitrite may function as an alternate electron acceptor.

Genes involved in fatty acid degradation exhibited increased expression under KCl-induced stress and to a lesser extent under NaCl-induced stress. Following NaCl exposure, the *prpR* regulatory gene showed increased expression, implying activation of the *prp*-encoded system that catabolizes the short-chain fatty acid propionate.

Finally, expression of the *asnA* and *asnB* genes suggests that exposure to both KCl and NaCl caused an increase in l-asparagine biosynthesis. NaCl-exposed cells also showed an increased level of *glnA* expression, which is involved in glutamine synthesis. Glycerol induced the upregulation of a number of histidine biosynthetic genes.

### (ii) Osmoadaptation after 1 h of exposure.

Several genes associated with osmoadaptation were downregulated following exposure to glycerol. These included the *proP*, *proU*, and *osmU* (STM1491 to STM1494) osmoprotectant transporter genes as well as genes for glutamate formation and trehalose biosynthesis (both via *otsAB* and conversion via glycogen). In contrast, at this time point the *putP* gene, encoding a proline permease, was upregulated under KCl-induced stress, as was the case for other transporters (*proV*, *osmY*) as well as *putP* under NaCl-induced stress. Expression of the *kdpE* response regulator of the KdpD-KdpE two-component system controlling the potassium transport operon was depressed upon addition of KCl.

### (iii) Membrane and motility changes after 1 h of exposure.

Extensive changes may be occurring in the structure of the cell membrane following glycerol-induced stress, with many genes associated with membrane formation, lipopolysaccharide (LPS) biosynthesis, O-antigen expression, and membrane stability being upregulated (see Data Set S3 in the supplemental material). The *tol-pal* system was also upregulated in the presence of glycerol (27). The upregulation of flagellar gene expression was also noted.

In contrast to the findings for samples exposed to glycerol, exposure to both KCl and NaCl caused the downregulation of a number of genes associated with LPS synthesis (see Data Sets S1 and S2 in the supplemental material). The transcriptional activator *rfaH*, required for full expression of the *rfb* and *rfa* operons, was downregulated by 3.8-fold in comparison to the control level following KCl-induced stress, while expression of *mdoC*, the gene responsible for the succinylation of osmoregulated periplasmic glucans, decreased by 60-fold (28). Similarly, four genes of each of the *rfa* (*rfaI*, *rfaJ*, *rfaY*, *rfaZ*) and *rfb* (*rfbN*, *rfbU*, *rfbV*, *rfbX*) loci showed decreased expression following NaCl-induced stress. At 1 h after NaCl exposure, the upregulation of the periplasmic negative regulator of the CpxRA system, *cpxP*, was noted. It was also observed that *adrA*, the regulator of cellulose formation, showed significant downregulation when cells were exposed to NaCl.

### (iv) Other stress response mechanisms observed after 1 h of exposure.

At 1 h, the phage shock genes *pspABCD* were upregulated after NaCl- and KCl-induced stress. In contrast, glycerol exposure reduced the expression of the regulator of this operon, *pspA*, at this time point.

The upregulation of putrescine production via the conversion of l-arginine by the *speA* and *speB* genes was observed in the presence of glycerol (although the downregulation of *speF* indicated that formation via ornithine was unnecessary).

### Identification of differentially expressed genes after 6 h of exposure to the three humectants.

**FIG 3:**
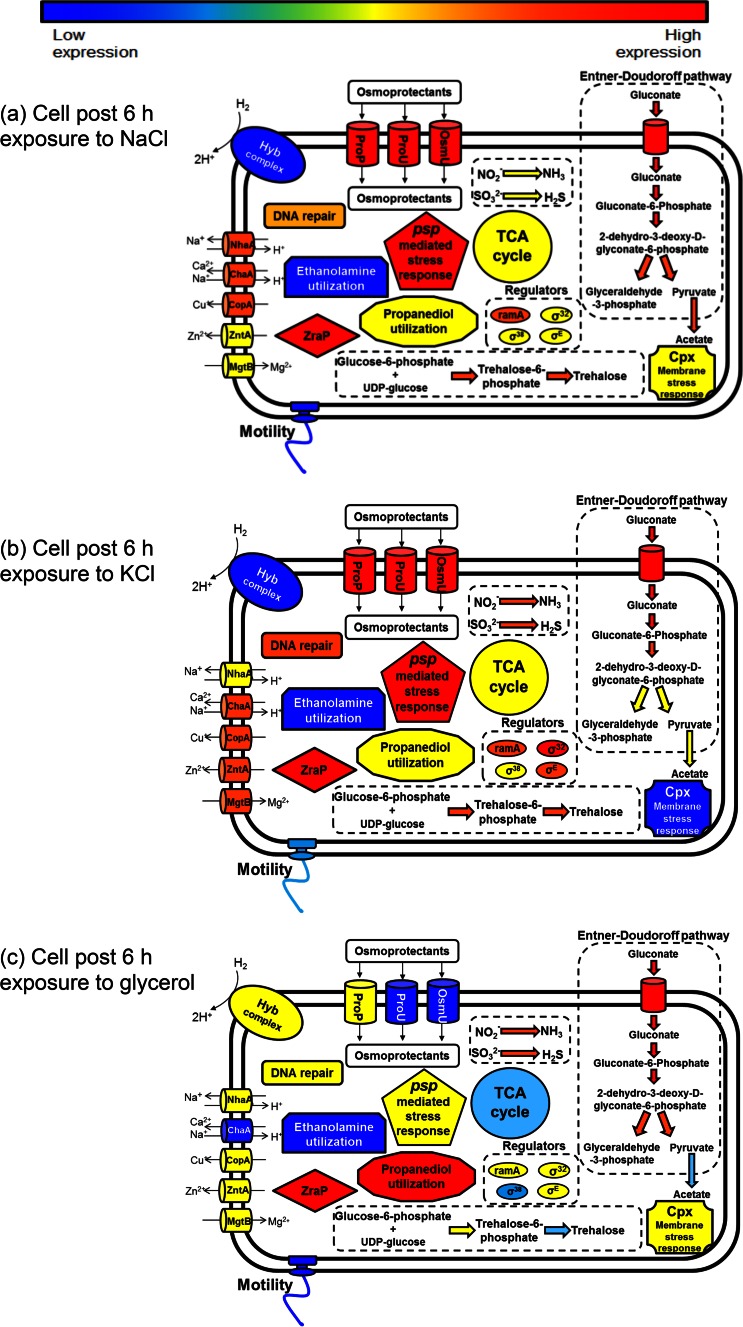
Summary of proposed changes occurring after 6 h of exposure to NaCl (a), KCl (b), or glycerol (c).

In contrast to the results obtained at 1 h after exposure to the three humectants, at 6 h after exposure, KCl-exposed cells showed the most differentially expressed genes according to the change in expression of greater than 3-fold (*P* < 0.05) compared with that of the ESP control, with 334 genes being upregulated and 409 genes being downregulated (total number of genes with changes in expression, 743) (Table 1). In the case of exposure of the strain to NaCl and glycerol, there were 481 and 450 differentially expressed genes, respectively. Figure 3 summarizes the changes in expression in the bacterial cell occurring at 6 h postexposure to humectants.

### (i) Central metabolism and energy derivation after 6 h of exposure.

After 6 h of exposure, samples exposed to all three humectants showed increased expression of genes encoding gluconate uptake and the degradative enzymes of the Entner-Doudoroff pathway. Glycerol-exposed cells appeared to also generate pyruvate via the *garL* gene, which imparts the ability to cleave 5-dehydro-4-deoxy-glucarate and 2-dehydro-3-deoxy-d-glucarate to yield tartronate semialdehyde and pyruvate, respectively. Similar to the observations for the samples at 1 h after exposure, the pyruvate formate lyase activator was upregulated following exposure to glycerol. In contrast, at this time point the repressor of the pyruvate dehydrogenase complex, *pdhR*, was upregulated following exposure to both KCl and NaCl. The NaCl-exposed samples also showed an increase in the expression of the *poxB* gene, which encodes the enzyme pyruvate dehydrogenase, which catalyzes the conversion of pyruvate to acetate.

In contrast to the findings at the 1-h time point, the effect of glycerol exposure on central metabolic pathways was somewhat reduced after 6 h, with the levels of many genes involved in the TCA cycle returning to control levels. At 6 h, the upregulation of *asrA* and *asrC*, two components of the anaerobic sulfite reductase (along with *asrB*), was noted after growth in glycerol. Some components of other alternative respiration systems, such as *nirB* (nitrite reductase) and *ttrB* (part of tetrathionate reductase), were also upregulated. Increases in the expression of *pdu* genes (which functions in propanediol utilization) and the associated regulator (*pocR*) were also noted in glycerol-exposed cells.

KCl-exposed cells displayed an increase in glutamine synthesis, while it was noted that ethanolamine utilization was downregulated under all conditions at this time point.

### (ii) Osmoadaptation after 6 h of exposure.

While NaCl- and KCl-exposed cells revealed the significant upregulation of osmoprotectant transport systems (*proP*, *proU*, *osmU*) and trehalose biosynthesis, the opposite effect was observed under glycerol-induced stress, which caused the decreased expression of all these processes. Similarly, the osmotically inducible protein encoded by *osmC* was upregulated following 6 h of exposure to NaCl, but the same gene was downregulated in glycerol-exposed samples. The *mngB* gene codes for the synthesis of the osmolyte mannosylglycerate and was upregulated only in NaCl-exposed samples (29).

### (iii) Membrane and motility changes after 6 h of exposure.

During glycerol exposure, the extensive gene expression changes related to the cell membrane and LPS synthesis appeared to have stabilized by 6 h, with the levels of regulation of the majority of these returning to control levels (see Data Set S3 in the supplemental material). However, the opposite observation was made following KCl treatment, which upregulated a number of genes linked to LPS and O-antigen synthesis, as well as the *wzzB* O-antigen chain length regulator. However, downregulation of the LPS core biosynthesis regulator *rfaH* (also downregulated in NaCl-exposed samples) and the *rcsB* and *yojN* (*rscD*) 2-component system was also noted. The *cpxP* repressor of the *cxpRA*-regulated envelope stress response was also upregulated in KCl-exposed bacteria.

A range of flagellar genes, including the *fliA* sigma factor, as well as a number of chemotaxis-associated genes was downregulated under all three conditions, consistent with a reduction in motility.

### (iv) Other stress response mechanisms observed after 6 h of exposure.

Heat shock protein-encoding genes *hslSTUV* and *htpG* and the phage shock *psp* operon were upregulated in the presence of both KCl and NaCl after 6 h of exposure. The *katE* and *sodC* genes were both upregulated in NaCl-exposed samples, indicating a response to oxidative stress within these cells; in contrast, *katE* was again downregulated in glycerol-exposed cells.

A number of DNA repair genes were upregulated in samples exposed to NaCl (*nfo*, *recN*) and KCl (*mutS*, *nei*, *nfo*). Similarly, the *groEL*, *groES*, and *dnaK* chaperone genes were also upregulated, perhaps reflecting the presence of misfolded proteins.

It was found that the *ramA* global regulatory gene was overexpressed after both KCl and NaCl exposure, while both *rpoE* and *rpoH* were upregulated by KCl exposure alone. Glycerol-exposed samples continued to show the downregulation of the *rpoS* sigma factor.

### (v) Inorganic ion transport and metabolism changes after 6 h of exposure.

Several genes involved in the transport of inorganic and transition metal ions were upregulated in KCl- and NaCl-exposed samples. These included the *chaA* Ca^2+^/H^+^ antiporter and the *copA* Cu(I) transport P-type ATPase. Also, NaCl-induced stress uniquely resulted in the upregulation of the Na^+^/H^+^ antiporter (*nhaA*) and its activator, *nhaR*. In contrast, the *zntA* and *mgtB* P-type ATPase-encoding genes were upregulated under KCl-induced stress alone.

### Identification of differentially expressed genes after 24 h of exposure to the three humectants.

**FIG 4:**
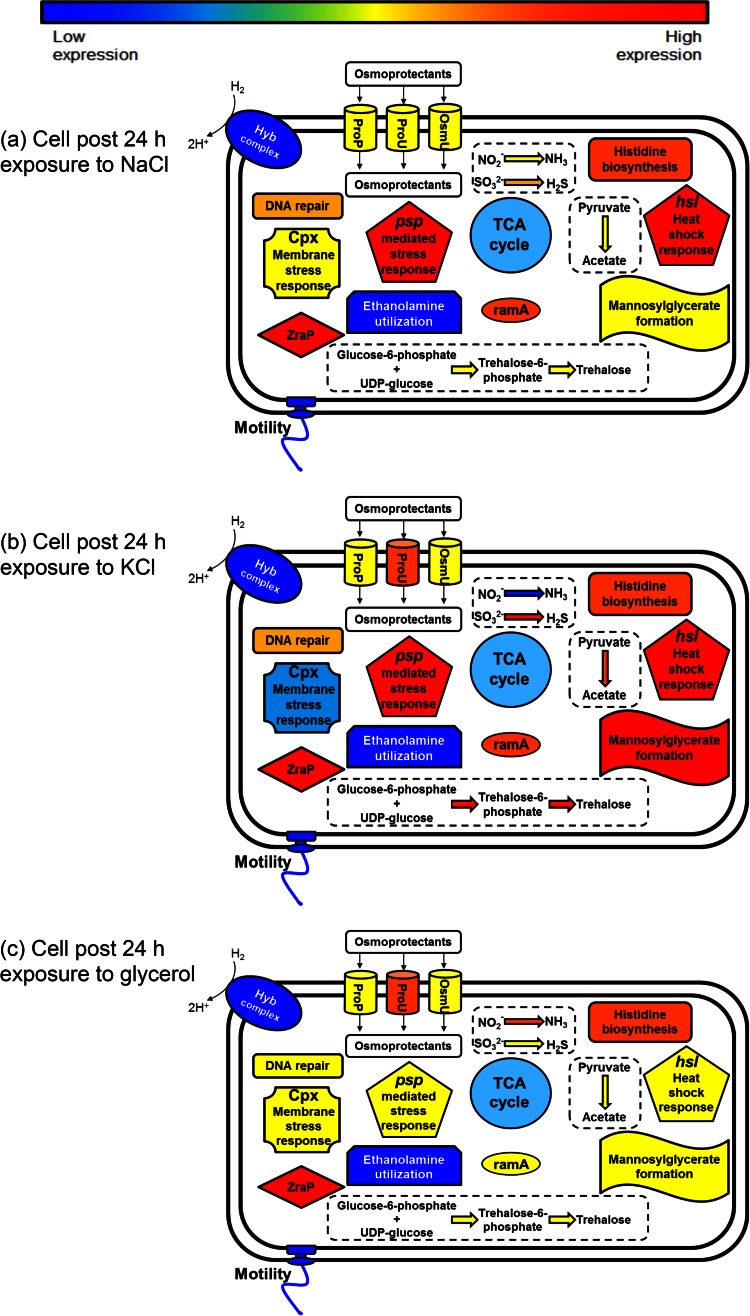
Summary of proposed changes occurring after 24 h of exposure to NaCl (a), KCl (b), or glycerol (c).

Following 24 h of exposure, cells were compared to the control inoculum in order to discern what overall changes occurred following inoculation. These results are described below and summarized in Table 1 and Fig. 4.

### (i) Central metabolism and energy derivation after 24 h of exposure.

Among the genes involved in the TCA cycle, downregulation was recorded for the isocitrate dehydrogenase-encoding gene (*icdA*) in both NaCl- and glycerol-exposed samples, while the malate dehydrogenase (*mdh*) gene showed a significant decrease across all three exposure conditions. The *poxB* gene, which catalyzes the conversion of pyruvate to acetate, was upregulated only in KCl-exposed samples. Genes involved in the Entner-Doudoroff pathway no longer showed any significant change in expression in cells exposed to the three humectants in comparison to their expression in the control inoculum.

Compared to the gene expression in the control inoculum, histidine biosynthetic genes were upregulated after 24 h of exposure to all three humectants. Conversely, the *eut* genes required for ethanolamine utilization were significantly downregulated in each sample.

### (ii) Osmoadaptation after 24 h of exposure.

After 24 h, only samples exposed to KCl showed any significant changes in the expression of genes related to osmoregulation compared to their expression in the control inoculum. These included the *proU* osmoprotectant transporter genes, the osmotically inducible *osmC* gene, and the *mngB* gene, responsible for the formation of the osmolyte mannosylglycerate. In KCl-exposed cells, the upregulation of genes involved in galactose metabolism and the subsequent conversion of galactose to UDP-glucose was noted. The formation of UDP-glucose also links in with the biosynthesis of the disaccharide trehalose, the gene for which was also upregulated.

### (iii) Membrane and motility changes after 24 h of exposure.

Similar to the data observed at an earlier time point, the *cpxP* repressor of the Cpx envelope stress response was upregulated by KCl exposure compared to its level of exposure in the control inoculum. However, contrary to the results obtained after 6 h of KCl exposure, after 24 h a number of genes involved in O-antigen expression and LPS biosynthesis were downregulated, as were genes for a number of outer membrane proteins (*nmpC*, *ompC*, *ompS*, *ompW*), when their expression was normalized to that of the ESP inoculum.

### (iv) Other stress responses observed after 24 h of exposure.

Similar to the findings at previous time points, after 24 h the *psp* phage shock genes showed a significant induction in the presence of both NaCl and KCl compared to that in the control inoculum culture. In KCl-exposed samples, the *mutM* gene, involved in DNA repair, as well as the *groEL*, *groES*, and *dnaJ* chaperone-encoding genes, which are responsible for preventing the aggregation of misfolded proteins under stress, were upregulated. Similarly, the *hslORSTUV* and *htpG* heat shock protein-encoding genes, as well as the *katE* and *sodC* genes highlighted above, were upregulated by more than 3-fold in the KCl-exposed samples.

Samples exposed to all three humectants showed the downregulation of genes responsible for flagellar assembly and chemotaxis (including the *fliA* sigma factor), comparable to the effects observed at the 6-h time point.

## DISCUSSION

In the context of the modern food industry, consumer awareness about the adverse health effects linked to a diet containing high levels of salt has resulted in pressure being placed on manufacturers to formulate low-*a_w_* food matrices that contain reduced amounts of NaCl. The humectant compounds KCl (an ionic humectant) and glycerol (a nonionic humectant) may offer an acceptable alternative and could thus be considered. Currently, little is known about the bacterial response to either KCl or glycerol.

In this investigation, the transcriptomic response of *S*. Typhimurium 4/74 following exposure to NaCl, KCl, and glycerol was studied. The response to NaCl-induced stress has been the main focus of investigations of osmoregulation in *S*. Typhimurium (14, 30, 31) and was included here to directly compare the transcriptomic responses across all three conditions.

Shabala et al. challenged the previously held view that bacteria respond to the stress imposed by all osmotic compounds in the same manner, for example, by inducing an initial increase of K^+^ influx, regardless of whether that compound is ionic or nonionic (13). That study investigated the response of E. coli to NaCl (ionic) and sucrose (nonionic) after a 10-min osmotic shock. The data indicated that only 57% of the same genes were upregulated after exposure to the two compounds (13). These data are generally supported by the findings of this study, insofar as NaCl and KCl (ionic compounds) induced changes in gene expression that were more comparable than the changes found in cells exposed to glycerol (nonionic) under the same *a_w_* conditions.

Previous studies have focused on the effect(s) of equal molar concentrations of ionic salts (rather than equal *a_w_* levels) to investigate their impact on the growth of Listeria monocytogenes (32, 33). These studies reported that different ionic compounds can have various bacteriostatic effects when applied at the same molar concentrations. However, Boziaris et al. demonstrated that although equal molar concentrations of NaCl and KCl have similar *a_w_* values, they appear to exert similar effects on growth (32). In this study, our focus was to investigate the transcriptional responses arising from the use of three humectants applied at the same *a_w_*, comparing the effects of two ionic compounds with the effect of a nonionic compound, glycerol.

A summary of the changes in gene expression occurring at each time point are broadly summarized in Fig. 2, 3, and 4. The key findings are discussed below.

### Alterations in metabolic pathways.

The transcriptomic data obtained in this study suggest that the addition of humectants gives rise to the upregulation of gluconate transport and the degradative components of the Entner-Doudoroff pathway responsible for catalyzing the interconversion of gluconate to form pyruvate and glyceraldehyde-3-phosphate. This upregulation occurred at an earlier time point in response to glycerol than the time of its occurrence in response to NaCl and KCl. These data suggest that the cells are switching to gluconate as their primary carbon source in response to glycerol. Interestingly, this pathway has also been found to be upregulated in *S*. Typhimurium during macrophage infection, and it is also necessary for E. coli colonization of the murine large intestine, suggesting a virulence-associated role (34, 35). In the current study, we show that induction of the Entner-Doudoroff pathway may also be linked to changes in the water activity of the environment. These enzymatic reactions may also provide the cell with reducing power in the form of NADPH, which can be utilized by other cellular reactions (36).

During glycerol-induced stress, it appears that cells may be reducing their oxygen consumption by upregulating alternative (anaerobic) respiration systems. This is reflected by the downregulation in the expression of genes encoding the enzymes of the TCA cycle and the concomitant upregulation of the pyruvate formate lyase activator enzyme. When active, pyruvate formate lyase catalyzes the nonoxidative conversion of pyruvate to formate and acetyl coenzyme, which can then act as a substrate for mixed acid fermentation. The downregulation of acetate formation by *poxB* was also observed. Similarly, if the cells shift to an anoxic metabolism format, alternative terminal electron acceptors are required in order to support the provision of cellular energy. In line with this hypothesis, over the course of the study it appeared that glycerol-exposed cells may have been using alternative respiration systems involving fumarate, nitrite, sulfite, and also, possibly, tetrathionate. However, other studies have reported that these systems can sometimes show elevated expression independently of whether the relative electron acceptors are present (37). This observation will require further investigation in the future. Similarly, in KCl- and NaCl-exposed cells, the corresponding components of the terminal electron transport chain exhibited signs of reduced activity after 6 h of incubation.

It is important to consider the fact that the metabolic shift to a more anoxic state that is observed may also result from the storage conditions rather than the humectants themselves. As stated above, conditions were chosen to simulate a probable storage environment of a food matrix; therefore, no aeration was used. Recently, Metris et al. demonstrated that E. coli experiences a switch from aerobic to fermentative metabolism in the presence of osmotic stress (38).

### Osmoprotection.

When bacteria are exposed to osmotic stress, cells need to rapidly respond in order to cope with and adjust to the changes in external osmolarity that are occurring. This ability to adapt is integral to prevent the loss of water from the bacterial cell and ultimately aid survival. A common method of adaptation for bacteria is the accumulation of osmoprotectant molecules, including proline and glycine betaine (4, 16, 39). These low-molecular-weight solutes prevent water loss from the cell and, due to their neutral charge, can be transported into the cell and accumulate to high concentrations without affecting cellular function (16). Previous reports have outlined the contributions of the ProP, ProU, and OsmU osmoprotectant transport systems to survival under NaCl-induced stress (14, 17, 18, 40–42). The upregulated transcription of *proP* and *proU* only 6 min after NaCl addition was reported previously (14). The data from NaCl-exposed samples obtained in this study support and further validate these models. However, under KCl-induced stress, the induction of all three transporters did not appear to be among the initial responses of these cells, as little increase in expression was observed at 1 h postexposure. In contrast, all were significantly upregulated after 6 h. This response of the bacterial cell to the osmotic conditions imposed by KCl begins to suggest that alternative osmoregulatory mechanisms may exist to cope with the initial stages of KCl exposure. In support of this observation, another gene coding for an alternative proline transporter, *putP*, showed significant upregulation at 1 h after KCl addition. However, this transporter is thought to transport proline only for use as a carbon or nitrogen source and may not play a role in osmoadaptation (43–46). As these studies concentrated on the response to NaCl, it is possible that the PutP transporter may have an osmoprotective role in the presence of KCl during the early stages of osmotic shock, which is a unique observation.

Conversely, glycerol induced the downregulation of *proP*, *proU*, and *osmU*. Although these cells were exposed to the same *a_w_* levels, these data highlight the differential responses occurring in relation to osmoadaptation in the presence of alternative humectants, in this case, ionic compounds versus a sugar alcohol.

Trehalose is another compatible solute commonly synthesized in response to an osmotic downshift (14, 39, 47, 48) and has also been found to play a role in desiccation tolerance (6, 8). As expected, the expression of the trehalose biosynthetic genes in response to both KCl- and NaCl-induced stress increased at 6 h postexposure. Once again, glycerol demonstrated an alternative effect, with trehalose-related gene expression being reduced at 1 and 6 h, again highlighting the unique features in the mechanisms of the response to these humectant compounds, even though water activity remained constant.

The upregulation of fatty acid catabolism was previously documented for Salmonella cells desiccated onto stainless steel, where it was postulated that this may occur because glucose is diverted into trehalose biosynthesis (6). As such, it was believed that the bacterial cell may switch to fatty acid catabolism to generate the energy needed for biological reactions. Both KCl and NaCl exposure induced a similar scenario, by which increased trehalose synthesis was coupled with an increase in the expression of *fad* genes, but only after 1 h of exposure.

Taken together, these data highlight the fact that *S*. Typhimurium 4/74 does not appear to respond to a reduction in water activity in a uniform manner, as the changes observed following exposure to glycerol did not follow traditional osmoadaptive response mechanisms.

### Membrane.

The ability of bacterial cells to stabilize and reinforce the membrane structure is key to survival under osmotic pressure. If the bacterial cell is unable to cope with the turgor pressure encountered, cell lysis and death will result. Changes in the expression of membrane-associated genes may be exerted in order to combat alterations in osmolarity due to the presence of glycerol and the associated reduction in intracellular water.

In this study, the addition of glycerol led to the significant upregulation of a large number of genes associated with LPS (*rfa* locus) and O-antigen (*rfb* gene cluster) biosynthesis as well as the *oafA* gene, required for the acetylation of the O antigen, as measured after 1 h of exposure (49–51). Linked with this, the Tol-Pal system, consisting of seven genes, *ybgC-tolQ-tolR-tolA-tolB-pal-ybgF*, also elicited significant increases in expression. This system appeared to have a variety of functions related to the stability and function of the outer membrane. It is also thought to be required for the import of group A colicins across the cell envelope (52). Aside from its role in membrane integrity, other functions of this system include the export of cell envelope proteins through the periplasm and the assembly of outer membrane proteins, and TolA has also been shown to play a role in the expression of the O antigen (27, 53–55). Gerding et al. reported that the Tol-Pal proteins have a role to play in cell division in E. coli, where they localize to the site of division and appear to promote proper invagination of the outer membrane (56).

The upregulation of the biosynthesis of putrescine was recorded after 1 h of exposure to glycerol. This divalent cation is essential for growth and exerts a number of functions within the cell. In regard to membrane and surface structures, this polyamine is one of the constituents of the outer membrane of Salmonella species (57, 58). It also plays a role in the regulation of the pore size of outer membrane proteins, including OmpF and OmpC, with binding of putrescine causing closure and a resulting decrease of outer membrane permeability (59).

The CpxRA system is an important factor controlling several genes in response to envelope stress (37). In terms of activation, this system is usually in an active state and is controlled by the activity of CpxP, which acts like a switch in order to turn off activity (37). The *cpxP* gene, coding for this periplasmic negative regulator, exhibited increased expression following exposure to both NaCl and KCl, suggesting that this particular envelope stress pathway is not required during osmotic challenge with these ionic compounds.

In contrast to the cellular effects observed following exposure to glycerol, 1 h of exposure to either NaCl or KCl resulted in a decrease in expression of genes associated with LPS biosynthesis. Conversely, after 6 h of exposure to KCl, a number of genes belonging to the *rfa* and *rfb* loci and the *wzzB* O-antigen length regulator were upregulated. However, decreases in the expression of the LPS core biosynthesis regulator (*rfaH*) and the *rcsB* and *yojN* (*rscD*) two-component system controlling capsule, cell division, and the expression of *wzz* genes were observed (60, 61). Deletion mutants of *S*. Typhimurium lacking the *rfaH* gene have been used as vaccine candidates in mice (61). As such, the decrease in expression of this regulator under NaCl- and KCl-induced stress may be associated with a decrease in the virulence of the pathogen. Overall, the effect and extent of membrane changes following KCl-induced stress remain unclear and may require further investigation.

In regard to membrane stress responses, both NaCl and KCl induced a significant upregulation of the *psp* genes at both 1 and 6 h postexposure. This system was first described in E. coli and was linked to filamentous phage infection (62). It has since been shown to play a role in the response to other stressors, such as ethanol, heat, and osmotic shock, where it is believed that the dissipation of the proton motive force (PMF) is an inducing signal (37, 63, 64). Induction of this system suggests that it may be the primary method of preservation of the membrane and the PMF when Salmonella cells are exposed to both NaCl and KCl.

The σ^E^ regulator, encoded by *rpoE*, showed a significant induction only under KCl-induced stress. This gene plays a pivotal role in the regulation of genes involved in the response to envelope stress, like that encountered under a variety of environmental stress conditions, such as heat, starvation, and osmotic shock with NaCl (65–68). Recent studies have also shown that this regulator is required for long-term desiccation survival (6, 7). Here, we demonstrate that this gene may also function in the response to envelope stress induced by KCl.

Overall, these data show that while the water activity has an effect on the cell membrane, the type of effect is highly dependent on the solute in question.

### Ion transport.

KCl-stressed bacteria also showed significant increases in the expression of a Zn^2+^ export gene, *zntA*. Similar to copper, zinc is also essential for growth but can become toxic at high levels, thereby leading to the induction of export pumps (69). Interestingly, in comparison to the findings for the control inoculum, all three humectants were shown to induce increases in the expression of *zraP*, a zinc resistance gene, after 6 and 24 h of exposure. ZraP is a membrane-bound protein that undergoes cleavage under high Zn^2+^ concentrations, leading to the release of the zinc-binding region into the periplasm (70). These results indicate that under these growth conditions, *zraP* may be important in sustaining viability.

### Other differentially expressed regulatory mechanisms.

Significant changes in the expression of a number of (global) regulatory genes were also observed. Interestingly, *ramA* was induced under NaCl- and KCl-induced stress but not in response to glycerol. Overexpression of this gene can lead to an increase in the level of resistance to certain antimicrobial compounds due to increased AcrAB-TolC expression and decreases in OmpF expression (71–73). Bailey et al. demonstrated that the involvement of the RamA regulon may not be restricted to antimicrobial resistance-related genes/mechanisms alone but may also play a role in virulence; however, this was not tested *in vivo* (73). Nonetheless, whether alterations in environmental *a_w_* levels exert an effect on the antibiotic resistance profile of Salmonella has not been described and may prove an interesting avenue for future investigation.

### Conclusions.

In conclusion, this study investigated the transcriptomic response of *S*. Typhimurium 4/74 to three humectants used in the modern food industry. It compared differential gene expression in response to all three compounds at a common water activity level. These data document the differential responses observed following exposure to ionic (NaCl, KCl) and nonionic (glycerol) water-reducing agents. In comparison to the differential gene expression achieved by exposure to NaCl and glycerol, cells exposed to KCl showed overall higher levels of differential gene expression. As many of these genes were of unknown function, further characterization may uncover specific survival mechanisms in response to KCl. After exposure to all three humectants, cells showed increases in gluconate metabolism and *zraP* expression. Cells exposed to NaCl and KCl demonstrated increases in osmoprotectant transport along with increases in the expression of the *ramA* and *psp* genes. In contrast, glycerol induced the downregulation of osmoprotectant transporters but gave rise to an initial increase in the expression of membrane-associated genes, perhaps indicating stabilization of the outer membrane.

Finally, the test conditions applied in this study were not typical of those used to completely inhibit growth, and it is also the case that organisms such as Salmonella would not be controlled in foods by preventing or slowing growth; complete elimination would be the goal. The significance of the work reported here should be considered in this context, and further work that builds on these findings should take account of this. Nevertheless, it is hoped that this information will allow food manufacturers to gain a deeper understanding of how foodborne microorganisms may respond when introduced to alternative water-limiting ingredients.

## Supplementary Material

Supplemental material
