# Supplementary material for: Exposure of Salmonella enterica Serovar Typhimurium to Three Humectants Used in the Food Industry Induces Different Osmoadaptation Systems
Source: Appl Environ Microbiol. 2015 Sep 4;81(19):6800–11. doi: 10.1128/AEM.01379-15 (PMC4561688; doi:10.1128/AEM.01379-15)
Supplement: Supplemental material [file supp_81_19_6800__index.html]

Supplemental material 

# Exposure of *Salmonella enterica* serovar Typhimurium to three humectants used in the food industry induces different osmo-adaptation systems

## Supplemental material

- Supplemental file 1 -

  Oligonucleotide primers used in qRT-PCR analysis (Table S1) and validation of microarray data using qRT-PCR (Fig. S1).

  PDF, 143K
- Supplemental file 2 -

  Gene changes occurring in *Salmonella* Typhimurium 4/74 after NaCl exposure (Data Set S1).

  XLSX, 107K
- Supplemental file 3 -

  Gene changes occurring in *Salmonella* Typhimurium 4/74 after KCl exposure (Data Set S2).

  XLSX, 172K
- Supplemental file 4 -

  Gene changes occurring in *Salmonella* Typhimurium 4/74 after glycerol exposure (Data Set S3).

  XLSX, 241K
