# Supplementary material for: Exposure of Salmonella enterica Serovar Typhimurium to Three Humectants Used in the Food Industry Induces Different Osmoadaptation Systems
Source: Appl Environ Microbiol. 2015 Sep 4;81(19):6800–11. doi: 10.1128/AEM.01379-15 (PMC4561688; doi:10.1128/AEM.01379-15)
Supplement: Supplemental material [file AEM.01379-15_zam999116622so1.pdf]

Table S1. Oligonucleotide primers used in qRT-PCR analysis

Gene	Forward Primer (5’ –> 3’)	Reverse Primer (5’-> 3’)
16S	ATTGACGTTACCCGCAGAAGA	GGGATTTCACATCCGACTTGA
pocR	CTCCGATTGCTGCGGATTGT	CTGACCCTGAGCTCTTGAATG
putP	GGTGTTCTGCTGTCTGCTATC	TACCACCAGCACCATCACTCG
ompW	CGACATAAGGACGCACTTTG	GTTGAATTGCTGGCGGCTACG
hisD	GAACGTCAACTGGCGGAACT	GTAATCGCATCCACCAAATCG
eutS	CAGCCTTCATCGCTAAATCG	GTCACGCTGGCGCATCTCA
virK	GATCCTCCTGTCCGAGGAACA	TGCCAACGGCGTATCATTGAA
fadL	GTCAGATTCCCAGCGACACC	ACTTCCGAGCGGTAGGTCAG
Edd	CACTTTACCCGAAGCGCCT	GATTGTGTCGTTGTCGTGCGT
zraP	GTCAGTAGCGCGTTGTATTC	ATGTGGCAACAGGGAGGTAG

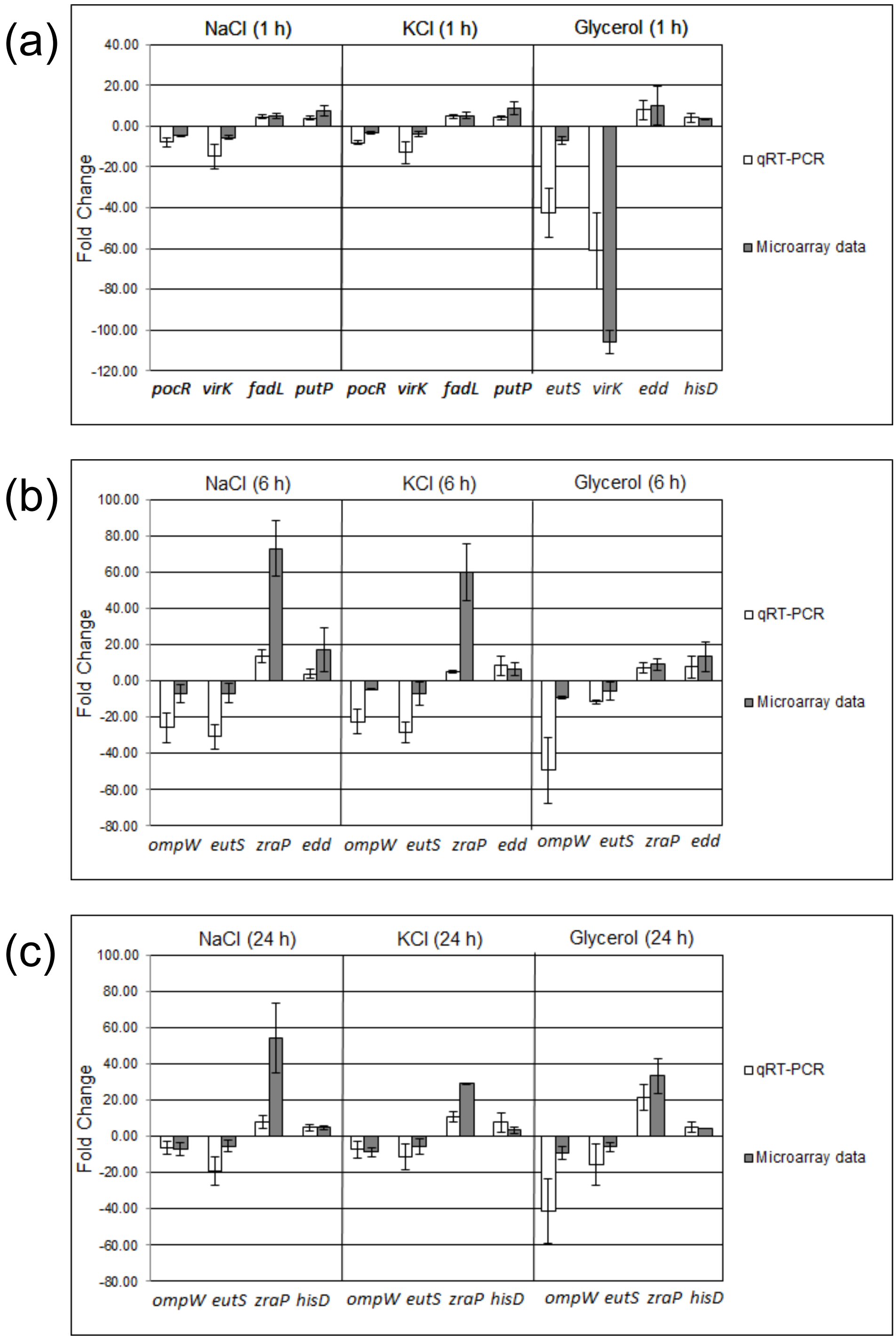

FIG. S1. Validation of microarray data using qRT-PCR at (a) 1 h, (b) 6 h and (c) 24 h. The 16S rDNA gene was used to normalise the data and fold-change was calculated using the $2^{-\Delta\Delta CT}$ method.
